# Graft-Versus-Host Disease Sustains Coagulation Activity for two Years After Pediatric Allogeneic Hematopoietic Stem Cell Transplantation

**DOI:** 10.1177/10760296241304771

**Published:** 2025-02-04

**Authors:** Satu Långström, Minna Koskenvuo, Pasi Huttunen, Riitta Lassila, Mervi Taskinen, Susanna Ranta, Markku Heikinheimo, Anne Mäkipernaa

**Affiliations:** 1 Division of Hematology-Oncology and Stem Cell Transplantation, Children's Hospital, University of Helsinki and Helsinki University Hospital, Helsinki, Finland; 2 Department of Pediatrics, Turku University Hospital, 8058 University of Turku, Turku, Finland; 3 Unit for Coagulation Disorders, Department of Hematology, Comprehensive Care Center and Cancer Center, Helsinki University Hospital, and Research Program Unit in Systems Oncology, 3835 University of Helsinki, Helsinki, Finland; 4 Astrid Lindgren Children's Hospital, Karolinska University Hospital and Karolinska Institute, Stockholm, Sweden; 5 Department of Pediatrics, Washington University in St. Louis, St. Louis, USA; 6 Faculty of Medicine and Health Technology, Center for Child, Adolescent, and Maternal Health Research, Tampere University, Tampere, Finland

**Keywords:** coagulation, pediatric, hematopoietic stem cell transplantation, graft-versus-host disease, endothelium

## Abstract

**Aim:**

To evaluate the longitudinal coagulation profile after allogeneic hematopoietic stem cell transplantation (HSCT) in pediatric patients with hematological malignancies.

**Methods:**

Several coagulation variables were measured at predetermined time points for two years after HSCT in 30 pediatric patients.

**Results:**

At six months post-HSCT, endothelial activation was reflected by 1.4-fold increase in circulating von Willebrand factor activity (p < 0.05), and by 2-fold increase in thrombin-antithrombin complex levels (p < 0.05), suggesting sustained coagulation system activity. In six patients with chronic graft-versus-host disease (cGVHD), specifically in those having gastrointestinal (GI) tract cGVHD, we observed continued longitudinal alterations in the coagulation system. The activities of both, coagulation factors (FV, FVII, FVIII, fibrinogen), and natural anticoagulants (antithrombin and protein C) were higher than prior to conditioning (p < 0.05) at most time points in patients with cGVHD. Moreover, fibrin turnover marker D-dimer was elevated from 6 to 18 months after HSCT (p < 0.05).

**Conclusion:**

Pediatric patients undergoing HSCT demonstrate prolonged derangement of the coagulation system, with a new alleviating balance after 6 months post-HSCT. However, in patients with cGVHD, and in particular when cGVHD affects the GI tract, the persisting derangement of coagulation suggest its contributing role in cGVHD and related complications.

## Introduction

Allogeneic hematopoietic stem cell transplantation (HSCT) is an effective treatment modality for pediatric patients with hematological malignancies.^1^ HSCT may, however, be followed by serious complications, which may cause long-lasting and significant morbidity. During the first months after HSCT, coagulation system may be disturbed due to conditioning regimen, infections, and many specific short-term complications of HSCT, such as sinusoidal obstruction syndrome of the liver (SOS/VOD), capillary leakage syndrome, engraftment syndrome, and thrombotic microangiopathy.^2–5^

Previously, we reported coagulation system disturbances during the first three months after HSCT in this same group of pediatric patients treated for hematological malignancies.^6^ The patients showed early coagulation activity, including endothelial damage and activation, enhanced fibrin turnover, and elevation of various coagulation factor activities. Altogether, those findings suggested a procoagulant state post-HSCT, which other studies have confirmed via observations on decreased natural anticoagulant functions as well as the inhibition of fibrinolysis after HSCT.^7,8^

Graft-versus-host disease (GVHD) remains a major complication of HSCT. Three major organs involved in acute GVHD (aGVHD) are skin, gastrointestinal (GI) tract and liver, whereas in chronic GVHD (cGVHD) the symptoms extend also to other organs, and the variable clinical features resemble other known autoimmune and immunologic conditions.^9^ Acute GVHD is traditionally considered to emerge during the first three months after HSCT, and cGVHD develops thereafter, though also cGVHD with aGVHD features has been characterized.^9^ Certain coagulation system abnormalities have been linked to the aGVHD in adults; these include endothelial dysfunction, increased thrombin generation, and increased activities of the natural anticoagulants antithrombin and protein C.^10–13^ Longitudinal studies on coagulation variables after the first three months post-HSCT are, however, scarce. In our previous study, no differences in coagulation variables between the pediatric patients with or without aGVHD were observed.^6^ However, both acute and chronic GVHD have been reported to increase the risk for hemostatic and thrombotic complications, both in adult and pediatric patients.^14–16^

We aimed to clarify the behaviour of coagulation factors and their regulators beyond the first three months after HSCT in pediatric patients with hematological malignancies, followed up for two years after allogeneic HSCT. We also assessed whether the coagulation status was associated with the cGHVD.

## Methods

### Patients

We prospectively enrolled 30 consecutive pediatric patients with hematological malignancies who underwent allogeneic HSCT between May 2012 and August 2016 in Helsinki University Hospital, in the Hospital for Children and Adolescents. A written informed consent was obtained from the parents or legal guardians of the patients, and from the patients older than 10 years. Institutional Ethics Committee approved the study.

### Laboratory Measurements

The blood samples were collected prior to the conditioning, and at 1, 3, 6, 12, 18, and 24 months after HSCT. Samples were not obtained after relapse. Blood samples were collected either from the central venous line or from the peripheral veins. If the central venous line was used, blood (2–5 ml) was drawn prior to the investigational sample to omit the effect of heparin, used to maintain the patency of the catheter. Blood was collected into citrated tubes, and immediately centrifuged at 2500 g for 15 min. Plasma was separated and stored at −70 °C.

Prothrombin fragment F1 + 2 (a specific measure of FXa activity leading to thrombin generation) and thrombin-antithrombin complexes (a measure of thrombin generation and its inhibition by antithrombin, TAT) were analysed with an Enzygnost F1 + 2 and Enzygnost TAT ELISA-based kits (Siemens, Healthcare Diagnostics, Marburg, Germany), respectively. Prothrombin time, fibrinogen, and FVIII were analysed with the BCS XP Coagulation analyser (Siemens Healthcare Diagnostics, Marburg, Germany) at the coagulation laboratory of Helsinki University Hospital (HUSLAB); PT (prothrombin time, %) with Nycotest PT reagent (Axis-Shield, Oslo, Norway), fibrinogen with the modification of the Clauss method (Multifibren U, Siemens Healthcare Diagnostics), and FVIII using Pathromtin SL and the specific coagulation factor-deficient plasma (Siemens Healthcare Diagnostics). Antithrombin and protein C activities were measured with chromogenic assays (Berichrom Antithrombin III and Berichrom Protein C), and D-dimer with an immunoturbidometric assay (Tina-quant D-dimer, Roche Diagnostics, Mannheim, Germany). FV and VII clotting activities were measured by BCS XP Coagulation analyser utilizing commercial factor-deficient plasmas (Siemens Healthcare Diagnostics, Germany). Von Willebrand factor (VWF) activity was assessed by BC von Willebrand Reagent or by Innovance VWF Ac (Siemens Healthcare Diagnostics), the methods having identical reference ranges and superimposable results.

### Statistical Analyses

Data are expressed as medians and interquartile ranges unless otherwise stated. Non-parametric tests were used for all comparisons. Mann Whitney *U*-test or Wilcoxon test were used for non-paired and paired comparisons, respectively. Data were analyzed using GraphPad Prism 5.0 (San Diego, CA; USA). A two-sided p-value <0.05 was considered statistically significant.

## Results

### Patient Characteristics

The study population consisted of 30 pediatric patients with hematological malignancies, acute lymphoblastic leukemia (ALL) being the most frequent underlying diagnosis (Table 1). All patients received myeloablative conditioning regimen. The patients who received a graft from an unrelated donor, received anti-thymocyte globulin (ATG) as a part of the conditioning regimen. The donors were an HLA-identical sibling, 10/10, and 9/10 HLA-match unrelated donor in 10, 18, and 2 patients, respectively. Cyclosporine was the most frequently used regimen to prevent GVHD, accompanied by methotrexate in those patients who received a graft from an unrelated donor (Table 1).

**Table 1.** Patient Characteristics.

Patients	n = 30
Age (years)*	
median(range)	10.5 (3–18)
*Sex*	
Male	19 (63%)
Female	11 (37%)
*Underlying diagnoses*	
ALL	19 (63%)
AML	6 (20%)
NHL	2 (7%)
MDS	2 (7%)
CML	1 (3%)
*Donor*	
MSD	10 (33%)
MUD	19 (63%)
CB	1 (3%)
*Type of graft*	
BM	29 (97%)
CB	1 (3%)
*Conditioning regimen*	
TBI-based	17 (57%)
Flu/Treo/TT	7 (23%)
Bu/Cy/Mel	4 (13%)
Other	2 (7%)
*Primary GVHD prophylaxis*	
Cyclosprorine	26 (87%)
* with MTX*	18 (60%)
MMF *with MTX*	3 (10%)
	
*Chronic GvHD*	6 (20%)
*Relapse during follow-up*	7 (23%)
*Death during follow-up*	4 (13%)

ALL, acute lymphoblastic leukaemia; AML, acute myeloid leukaemia; NHL, non-Hodgkin lymphoma; MDS, myelodysplastic syndrome; CML, chronic myeloid leukaemia; MSD, matched sibling donor; MUD, matched unrelated donor; CB, cord blood; BM, bone marrow; TBI, total body irradiation; Flu, fludarabine; Treo, treosulphane; TT, thiotepa; Bu, busulfan; Cy, cyclophosphamide; Mel, melphalan; GVHD, graft-versus- host-disease; MTX, methotrexate; MMF, mycophenolate mofetil *at the time of HSCT.

We evaluated thrombotic complications prior to and following HSCT in all children included in the study. Four patients had venous thrombosis prior to HSCT (three catheter-related thromboss and one cerebral venous sinus thrombosis), and they were treated with prophylactic doses of enoxaparin for 2 months, 9 months, or throughout the follow-up period (two patients). During the follow-up, one new thrombosis, related to central venous line, was observed.

Acute GVHD developed in 18 patients. Chronic GVHD was diagnosed in 6 patients during the follow-up period, and all of them had a preceding aGVHD. According to widely accepted classification^17^ cGVHD was mild in one patient (primary organ skin), moderate in one patient (primary was organ GI tract), and severe in four patients (primary organ was GI tract in three patients, and skin in one patient). In four patients, cGVHD was active and extensive during the whole 2-year period, requiring continuous treatment during the follow-up period. In two patients, cGVHD symptoms were alleviated earlier, after 6 and 9 months.

Four patients died during the follow-up period of two years, one patient of Epstein-Barr virus infection, one of respiratory syncytial virus infection, and two of relapse of the primary disease. Altogether seven patients relapsed during the study period (Table 1).

### Thrombin Generation and Fibrin Turnover from 6 to 12 Months After HSCT

**Figure 1.**
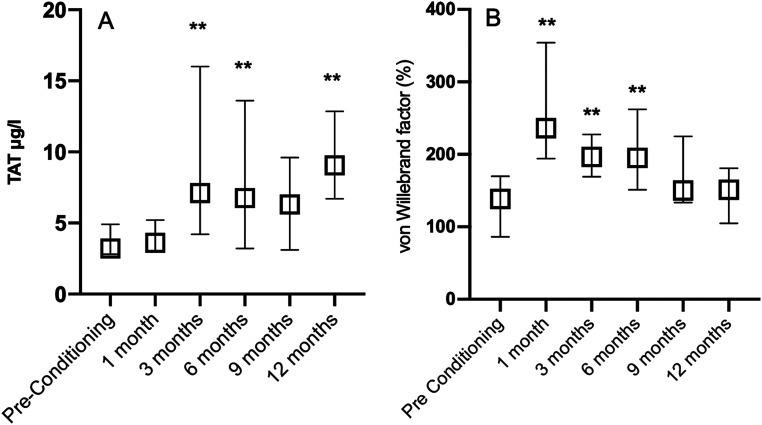
Thrombin-antithrombin complexes (TAT) (A) and von Willebrand factor (VWF) activity (B) prior to conditioning treatment of allogeneic hematopoietic stem cell transplantation (HSCT) and at 1, 3, 6, 9, and 12 months after HSCT. **p < 0.001 when compared with the Pre-Conditioning levels. Levels prior to conditioning, and at 1 and 3 months after HSCT have been presented previously.^6^ Data are presented as medians with intequartile range.

Of the variables measuring activation of coagulation, F1 + 2 or D-dimer did not alter during the 6 to 12 months period after HSCT when compared with the levels measured before the conditioning (Table 2). However, TAT levels on average doubled (p < 0.05) at 6 and 12 months after HSCT when compared with the pre-conditioning levels (Table 2, Figure 1).

**Figure 2.**
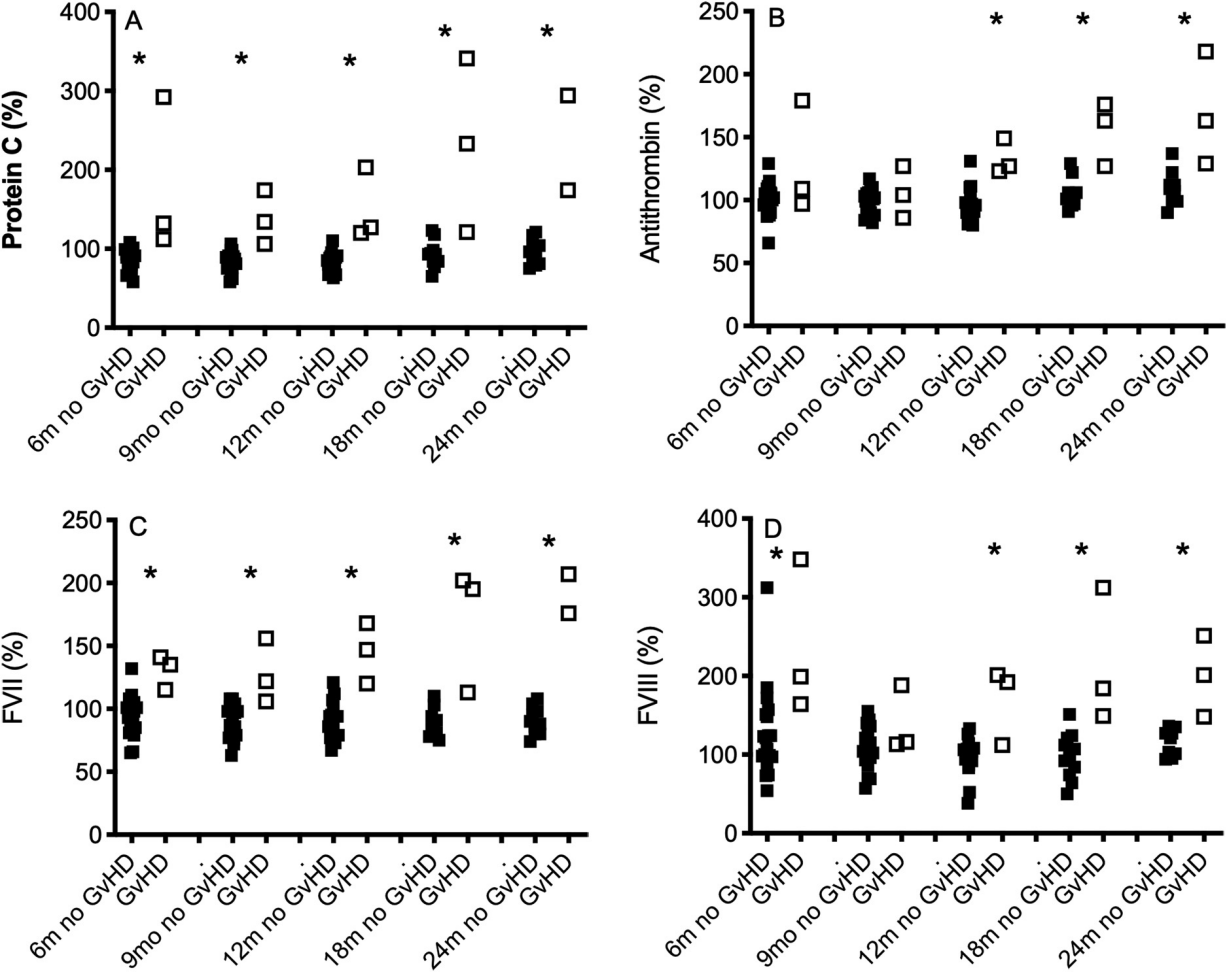
Activities of protein C (A), antithrombin (B), and coagulation factors VII (C), and VIII (D) during the 24-month period after allogeneic hematopoietic stem cell transplantation in pediatric patients with or without chronic graft-versus host disease (cGVHD) of the gastrointestinal tract. The coagulation and its upregulation were marked in the patients with cGVHD (open squares) of the GI tract when compared to the patients without cGVHD (black squares), *p < 0.05.

**Table 2.** Coagulation Variables.

	Pre-Conditioning	6 months	9 months	12 months	18 months	24 months
	(n = 30)	(n = 24)	(n = 24)	(n = 24)	(n = 15)	(n = 15)
F1 + 2 (pmol/l)	153 (71–391)	148 (91–372)	146 (77–798)	162 (104–1235)	na	na
TAT (ug/l)	3.2 (2.1–19)	6.8 (1.5–39)**	6.3 (0.7–50)	9 (1.5–19)**	na	na
D-dimer (mg/l)	0.3 (0.2–5.3)	0.3 (0.3–1.4)	0.3 (0.3–1.6)	0.3 (0.3–1.9)	0.3 (0.3–1.2)	0.3 (0.3–0.6)
PT (%)	81 (46–127)	87 (64–132)	92 (67–128)	91 (67–150)	94 (74–170)	110 (71–170)**
APTT (s)	28 (22–40)	31 (25–37)**	31 (26–37)**	32 (28–38)**	31 (27–42)	31 (27–46)
Thrombin time (s)	18 (15–24)	22 (17–43)**	21 (19–29)**	21 (18–33)**	20 (18–24)**	21 (18–23)*
Antithrombin (%)	101 (53–136)	103 (66–179)	100 (62–127)	92 (80–149)	103 (88–176)	111 (87–218)
Protein C (%)	84 (53–156)	92 (56–292)	88 (58–174)	85 (63–203)	94 (65–341)	100 (75–294)*
von Willebrand factor (%)	139 (63–324)	195 (71–496)**	150 (65–390)	151 (71–387)	154 (72–375)	136 (65–240)
Fibrinogen (g/l)	2.9 (1.4–4.1)	2.7 (1.8–5.0)	2.8 (2.0–4.9)	3.0 (2.1–4.9)	3.0 (2.2–9.0)*	3.2 (2.2–7.2)
Factor V (%)	94 (44–139)	111 (68–203)	105 (60–128)	100 (48–193)	113 (81–184)	108 (77–158)
Factor VII (%)	94 (56–146)	98 (56–141)	95 (63–156)	94 (67–168)	91 (75–202)	97 (74–207)
Factor VIII (%)	888–320)	124 (54–348)**	113 (57–188)**	104 (38–201)	107 (50–312)	124 (91–251)

* < 0.05, **p < 0.001, compared with the Pre-Conditioning level; median (range).

### Coagulation Screening Tests, Coagulation Factors, and Physiological Anticoagulants During 24 Months After HSCT

Coagulation screening tests, and the activities of physiological anticoagulants and coagulation factors were compared with the respective values measured prior to the conditioning. Prothrombin time (PT, %) and APTT remained mostly stable during the observation period from 6 months post-HSCT up to 24 months post-HSCT (Table 2).

Of the physiological anticoagulants, median activities of antithrombin and protein C were within the adult reference range throughout the study period, except for an increase of protein C activity at 24 months post-HSCT (Table 2).

Fibrinogen, FV, FVII, and FVIII were quite stable during the study period, FVIII being lower at 6 and 9 months after HSCT than before conditioning. However, VWF activity increased 1.4-fold at 6 months when compared with pre-conditioning activities (Table 2, Figure 1).

### Chronic GVHD and Coagulation Activity

To evaluate the possible association of the cGVHD and the coagulation system, we compared the coagulation variables of the six patients who had cGVHD with the patients without cGHVD. Of the natural anticoagulants, protein C was elevated (p < 0.05) early at 6 and 9 months post-HSCT, compatible with the timing of the increase of VWF activity. Also, FV was elevated in patients with cGVHD at 6 and 9 months, but FVII rose later from 12 to 24 months, as well as FVIII from 12 to 18 months, and fibrinogen at 12 months. D-dimer was elevated from 6 to 18 months (p < 0.05 for all comparisons).

We further evaluated those three patients who had cGVHD of the GI tract, and available blood samples covering the whole follow-up period of 24 months and compared them with the patients who did not have cGVHD. In these patients vWF was elevated at 6 months, FV from 6 to 12 months, FVII at all the time points, FVIII from 6 to 24 months, and fibrinogen from 12 to 24 months. Of the natural anticoagulants protein C was elevated at all the measured time points, and antithrombin from 12 to 24 months after HSCT. D-dimer was elevated from 6 to 18 months after HSCT (p < 0.05 for all comparisons) (Figure 2).

## Discussion

Coagulation system can be disturbed by various mechanisms after allogeneic HSCT. Previous studies, including our own, describe the coagulation system during the first three months post-HSCT,^6–8,10,12^ but the information on the later evolution of the coagulation activity after the first three months post-HSCT is scarce. Therefore, we followed these pediatric patients for a longer period, for up to 24 months post-HSCT.

Activation of the coagulation system could still be observed at six months after HSCT. At that time, TAT was increased, compatible with ongoing thrombin generation, and it remained elevated for at least up to 12 months after HSCT. With the same timing VWF, reflecting the activation and damage of the endothelium, was also increased at six months after HSCT. Endothelial damage is a well-known phenomenon after HSCT, and there are many specific HSCT-related conditions associating with the consequences of endothelial damage, eg sinusoidal obstruction syndrome of the liver (SOS/VOD), capillary leakage syndrome, and thrombotic microangiopathy.^2^ Endothelial damage has also been linked to the development of cGVHD, especially in patients who are refractory to steroids.^13,18^ Furthermore, the importance of endothelium has been highlighted in cGVHD studies in which an endothelial anticoagulant cofactor thrombomodulin has been reported to prevent cGHVD, when administered as a soluble recombinant regimen.^19^

The other measured coagulation factors and natural anticoagulants were mostly within reference range at six months post-HSCT and thereafter. Previous studies in pediatric patients by us and others on coagulation activity during the first months after HSCT showed that the procoagulant state prevailed with enhanced thrombin generation, elevated levels of coagulation factors, but attenuated levels of natural anticoagulants.^6–8^ After six months post-HSCT, the coagulation system seemed, however, to have reached steady state in most patients. Compatible with the clinical course in majority of the pediatric patients after HSCT, also most short-term complications of HSCT have already been alleviated at six months post-HSCT.

Patients with cGVHD suffer from severe long-term complications after allogeneic HSCT. Previously, in pediatric patients with ALL, cGVHD developed in 18–29% of the patients post-HCST, and further, extensive GVHD developed in 6–14% of the patients.^1^ In this study, cGVHD developed in 20% of the patients, and was severe and required treatment during the whole follow-up period in 13% of the patients. Since thrombotic and hemorrhagic complications typically emerge in patients with GVHD,^14–16^ we longitudinally evaluated the coagulation system especially in them.

In patients with cGVHD several abnormalitites in coagulation factor and natural anticoagulant activities were observed in comparison to the patients without cGVHD. These differences were more striking in patients who had active cGVHD of the GI tract. Elevated D-dimer in these patients suggested sustained fibrin formation, turnover and activation of fibrinolysis. FVIII and fibrinogen were also increased in patients with cGVHD at most time points during the follow-up period, aligning with their known properties as acute phase reactants.^20,21^ Also FV, whose role under inflammatory conditions has also been suggested,^22,23^ was elevated. All in all, these thrombo-inflammatory findings suggest ongoing coagulation and fibrinolytic activity in cGVHD patients.

Natural anticoagulants antithrombin and protein C were also elevated in patients with GI tract involvement. Protein C is known for its joint anticoagulant and anti-inflammatory properties.^24^ Recently, the importance of anti-inflammatory and cytoprotective activities of activated protein C in cGVHD was shown,^12,25^ and treatment with activated protein C was suggested.^25^

The impact of anti-thymocyte globulin (ATG) on coagulation has been a subject of debate. Some studies have suggested that ATG may induce a hypercoagulable state through mechanisms that link coagulation and complement activation.^26^ However, another study found no significant effect of ATG on the same coagulation variables that were also measured in the current study.^27^ Since any potential impact of ATG is likely to occur shortly after its administration, and our study focused on long-term effects on coagulation, the short-term effects of ATG were not assessed. Also, all but three patients had cyclosporin as primary GVHD prophylaxis; therefore, we were not able to compare the impact of cyclosporin on the coagulation status with other GVHD prophylaxis.

Limited number of patients in the current study poses a challenge to establish clinical phenomena. However, the studied patient group was homogenous; all were pediatric patients with a hematological malignancy. Further, though there were only three patients with cGHVD of the GI tract, with a complete follow-up data, the findings in their coagulation system were pronounced and uniform.

While previous studies mainly concentrate on the immediate post-HSCT-period, the patients were followed here for a longer period, and as expected, the turmoil in the coagulation system was calmed down after six months post-HSCT in majority of the patients depending on their clinical situation. However, in patients who suffer most from long-term complications of allogeneic HSCT, eg those with cGVHD, coagulation activity and imbalance between pro- and anticoagulant functions prevails for a longer period of time. The mechanisms underlying the elevated activities of the coagulation factors and anticoagulants refer to an interplay between coagulation and inflammatory pathways. More patients with cGVHD need to be studied on the hemostatic system and its regulation to reveal the reciprocal mechanisms on coagulation variables in the inflammatory milieu occurring in cGHVD, and further, these studies could support implementation of novel treatment options for thrombo-inflammatory conditions.
